# Supplementary material for: Brief psychological intervention for the prevention of deliberate self-poisoning: A randomized controlled trial from Sri Lanka
Source: PLOS Ment Health. 2026 Feb 20;3(2):e0000562. doi: 10.1371/journal.pmen.0000562 (PMC12922979; doi:10.1371/journal.pmen.0000562)
Supplement: S1 Appendix — (DOCX) [file pmen.0000562.s001.docx]

**Summary of the most frequently reported responses from thematic analysis**

| Theme | Description | Reported By |
| --- | --- | --- |
| Facing life's challenges: Ways of coping following the self-harm attempt |  |  |
| - *Seeking social support* | Participants talked to parents, friends, or close associates to get advice or emotional support. | Mainly younger participants; more prominent in the (brief psychological intervention) BPI group |
| - *Adaptive coping/acceptance* | Enduring problems by thinking about responsibilities (e.g., children) or adapting to circumstances. | Married women (mostly older) |
| - *Expressing negative emotions* | Venting anger or self-harming when stressed or frustrated. | Both the BPI and treatment-as-usual (TAU) groups |
| - *Self-distraction / activities* | Engaging in enjoyable or distracting activities such as music, TV, movies, or gardening. | Mostly the BPI group |
| Mechanisms of change | changes in the quality of relationships with close others -experiencing improved interactions with family or friends  Reflection after hospital stay, talking with peers who had similar experiences, and learning from the BPI intervention. | Mainly, younger participants in both the BPI and the TAU group  Mostly the BPI group |
| Attitudes toward BPI | Relief and emotional release from talking to a trained nurse; some retention of take-home calendar messages. | BPI group |
| Suggestions for improvement | Involving significant others in sessions; educating family members or spouses about coping and support. | BPI group |

Brief Psychological Intervention Group- BPI group

Treatment as usual group- TAU group
