# Supplementary material for: Brief psychological intervention for the prevention of deliberate self-poisoning: A randomized controlled trial from Sri Lanka
Source: PLOS Ment Health. 2026 Feb 20;3(2):e0000562. doi: 10.1371/journal.pmen.0000562 (PMC12922979; doi:10.1371/journal.pmen.0000562)
Supplement: Attachment — Submitted filename: Response to Reviewers.docx [file pmen.0000562.s005.docx]

**Response to Reviewers**

**Manuscript Title:** Brief psychological intervention for the prevention of deliberate self-poisoning: A randomized controlled trial from Sri Lanka

**Manuscript ID:** PMEN-D-25-00315

Dear Editor and Reviewers,

We sincerely thank the Editor and the reviewers for their careful evaluation of our manuscript and for their constructive and insightful comments. We have carefully considered all suggestions and revised the manuscript accordingly. We believe that these revisions have significantly improved the clarity, quality, and rigor of the paper.

Below, we provide a detailed, point-by-point response to each comment raised by the Editor and the reviewers. For clarity, reviewer comments are reproduced in black, our responses are provided in blue, and all changes made to the manuscript are highlighted in red in the revised version.

**Reviewer #1:** Interesting paper. Relatively large sample size. The effect is modest but the intervention is very cheap. The study may inspire to similar interventions in other countries. Many were lost during follow-up. How come?

**Response:**
We appreciate this observation. As we discussed in the paper, the high attrition is consistent with other longitudinal studies involving individuals who have recently self-harmed in Sri Lanka. Several contextual factors contributed to this loss during the follow-up. One factor might be that the majority of the study participants were young (16–25 years) and highly mobile, often relocating for work and education. We found that most of the participants provided temporary contact information; they had changed their contact information, which resulted in challenges in tracing them at 6 and 12 months. Another reason could be the stigma of being recontacted about a self-harm episode, which might also contribute to non-response. Further, a considerable proportion of the sample screened positive for depression at baseline, and individuals with depression are known to have lower follow-up rates in RCTs. We acknowledge this limitation in the Discussion and emphasise that the sample size was originally inflated to account for attrition. These revisions can be found on page 30, lines 613–617 of the tracked-changes manuscript.

**Reviewer #2:** This was a mixed method design study which the investigators labeled as a pilot randomized controlled trial (RCT) with embedded qualitative methods. The quantitative requirement of sample size was based on the continuous t-test format. However, the study analysis appeared to be based mostly on categorization of some of the major endpoints, depression, AUDIT and anxiety.

1. The protocol (Statistical Consideration) sample size is based on proportions. Please reconcile this.

**Response:**

Thank you for pointing this out. The sample size for our study was based on continuous PHQ-9 scores, calculated using a t-test to detect a clinically meaningful difference between groups, consistent with our protocol and prior studies. Accordingly, our primary analyses compared mean continuous scores of PHQ-9, GAD-7, and AUDIT between intervention and control groups (Table 5).

We also conducted categorical analyses to aid clinical interpretation, especially given the limited follow-up numbers. Trends in the categorical outcomes generally aligned with the continuous results, though they were not identical. Both approaches are now clearly described in the manuscript, with continuous analyses reflecting the primary outcome and sample size rationale, and categorical analyses included for feasibility and interpretation (pages 12–13, lines 247–276; page 17, lines 345–347; Table 5, page 18).

2. What was the rationale for categorization of some of the endpoints?
The analysis could have been more sophisticated using the continuous endpoints. There were two interventions and three time points. There is a loss of information with categorization as the authors should know.

**Response:**

We appreciate this comment. While continuous analyses provide greater statistical power, some outcomes in this study are inherently categorical (e.g., repeat self-harm episodes, suicidal ideation). Other measures, PHQ-9, GAD-7, AUDIT, and Brief COPE are originally continuous but were re-categorized based on established clinical thresholds to maintain adequate group sizes given the limited follow-up and to align with the pre-specified primary categorical outcomes of this pilot trial (Quantitative Data Analysis, pages 12–13, lines 247–281).

Continuous analyses were also conducted, and patterns generally aligned with those observed in categorical analyses. Categorization facilitates clinical interpretability while maintaining analytic feasibility. We acknowledge that this approach reduces sensitivity and may underestimate effect sizes, which is explicitly noted as a limitation in the Strengths and Limitations section of the revised manuscript (page 31, lines 620–625). The use of more sophisticated analyses (e.g., repeated-measures models) is acknowledged as appropriate for future full-scale trials.

3. Why not a repeated measures design with covariate adjustment, if needed?
As noted above, there seems to be somewhat of a disconnect between the protocol in the supplement and the text in the paper.

**Response:**

We fully agree that a repeated-measures design with covariate adjustment would be appropriate for a full-scale trial. However, this study was a pilot feasibility trial, and the analysis plan focused on simple group comparisons rather than modeling changes across multiple timepoints. The use of more sophisticated methods, such as repeated-measures analysis, is acknowledged as appropriate for a future full-scale trial in the Quantitative Data Analysis section, pages 13, lines 277–281.

4. The efficacy review in the protocol (11.3.2) mentions the relative risk (RR). Where is that?

**Response:**

Thank you for the comment. This is an omission we will correct. RR estimates were originally calculated but removed during the restructuring of the results section to improve clarity and flow. We have now re-inserted the RR values for the categorical outcomes in the Results section and added them to Tables 04 and 06 in the revised manuscript.

5. The authors mention attrition as a limitation, but talk about imputation in the protocol? What was the intended imputation? Was it explored?

**Response:**

Thank you for these comments. As noted in the protocol, multiple imputation was planned if missing data exceeded 20%. However, for the analyses presented in this manuscript, imputation was not employed. This decision was based on the attrition pattern, which was non-random (e.g., younger participants were disproportionately lost), and the presence of entire blocks of missing follow-up data for many individuals. Applying multiple imputation under these circumstances could have led to biased results; therefore, all analyses were conducted using complete-case data. We have clarified this in the Quantitative Data Analysis section (page 13, lines 272–275) of the revised manuscript.

6. There may be some typos in the paper. Please check for those.

For example, Table 4, six month GAD-7 for BPI the percent (88.4 + 7.6) does not add to 100, unless this reviewer missed something.

**Response:**

We appreciate this careful reading. This is indeed a typographical error. The cell labelled 7.6% should read 11.6%, which aligns correctly with the denominator. This has now been corrected in the revised Table 4 (page 17).

7. The qualitative information is somewhat informative. Some tabulations of the more common suggestion or responses noted might be helpful.

**Response:**
Thank you for this suggestion. In the revised manuscript, we have included a brief descriptive table summarising the most frequently reported common responses derived from the existing thematic analysis. This table has been added as a separate Appendix (S3 Appendix) to provide a concise overview of the common coping strategies and participant experiences identified in the qualitative interviews.

8. There was a lot of effort put into this important project by the investigators. The clarifications requested above would be helpful?

**Response:**

We appreciate both reviewers’ valuable comments. Their suggestions have significantly strengthened our manuscript. All requested clarifications have been incorporated into the revised submission.

We hope that the revisions satisfactorily address all concerns raised by the Editor and reviewers. We are grateful for the insightful feedback and the opportunity to improve our manuscript.

Sencerly,

Lakmini De Silva
